# An Unusual Presentation of Syncope in an Elderly Male Leading to a Diagnosis of an Anomalous Right Coronary Artery

**DOI:** 10.7759/cureus.48475

**Published:** 2023-11-07

**Authors:** Boris Avizov, Galib Mirza, Raed Bargout

**Affiliations:** 1 Internal Medicine, State University of New York Downstate Medical Center, Brooklyn, USA; 2 Osteopathic Medicine, Midwestern University Arizona College of Osteopathic Medicine, Glendale, USA; 3 Internal Medicine, Creighton University School of Medicine, St. Joseph’s Hospital and Medical Center, Phoenix, USA; 4 Cardiology, CHA Hollywood Presbyterian Medical Center, Los Angeles, USA

**Keywords:** anomalous coronary artery, right coronary artery anomaly, rare heart defect, recurrent syncope, congenital heart defect, cardiothoracic and vascular surgery, cardiology imaging, unusual cause of recurrent syncope, cardiology research

## Abstract

Various coronary artery anomalies have been identified in modern literature with most being benign in nature. Generally, these anomalous vessels are clinically silent due to their non-obstructive or benign course. It is vital to identify patients with malignant courses of these vessels as their initial presenting symptom might be sudden cardiac death. A 74-year-old male presented to the hospital following an episode of syncope and incontinence. Denying any symptoms of chest pain or shortness of breath, the patient did admit to having a six-month history of intermittent lightheadedness and one prior episode of syncope that was attributed to physical activity. Cardiac nuclear stress testing revealed a large reversible inferior wall defect indicating a defect with the right coronary artery. Cardiac catheterization demonstrated a history of coronary artery disease and revealed an anomalous origin of the right coronary artery. A coronary CT angiogram identified the right coronary artery as having an abnormal origin from the left sinus of Valsalva with a malignant interarterial route. The patient underwent a coronary artery bypass graft to correct the issue. There were no major postoperative complications. Treatment guidelines for patients suffering from malignant coronary artery anomalies are limited. Despite multiple surgical interventions available, data regarding conservative medical management is limited and should be of consideration in future studies.

## Introduction

Various coronary artery anomalies have been identified in modern literature with most being benign in nature. It is vital to identify patients with malignant courses of these vessels as their initial presenting symptom might be sudden cardiac death (SCD). We report the case of a 74-year-old African American man who presented with recurrent episodes of syncope and was found to have a malignant (interarterial) course of his right coronary artery (RCA) between his aorta and right ventricular outflow tract (RVOT).

## Case presentation

A 74-year-old African American male with a past medical history significant for type 2 diabetes, hypertension, and hyperlipidemia presented to the emergency department following an episode of syncope. The event occurred after the patient finished eating dinner with his family on New Year’s Eve. He admitted to having several alcoholic drinks along with his dinner. Family members also stated that they witnessed the patient having stool and urine incontinence lasting up to one minute during the syncopal episode. Upon arrival of the paramedics, he was found to have low systolic blood pressure in the 90s and two episodes of non-bloody, non-bilious emesis. He admitted to having prior episodes of lightheadedness and a single episode of syncope that occurred upon performing strenuous physical activity six months prior. While hospitalized, the patient denied having any residual effects of the event including trauma, seizures, chest pain, shortness of breath, or any other associated symptoms.

**Figure 1:**
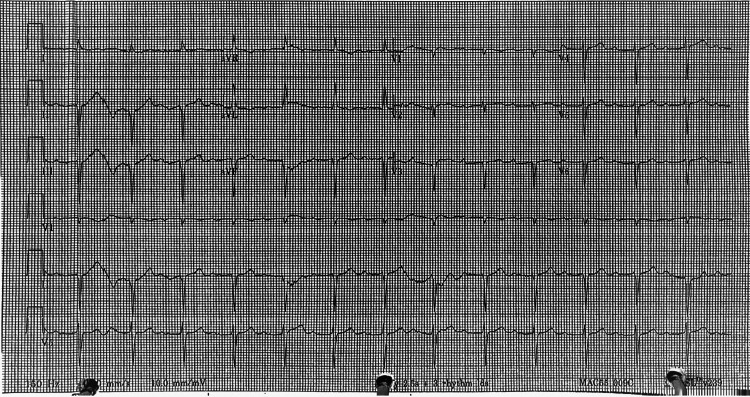
Electrocardiogram on admission was significant for a first-degree atrioventricular block with a PR interval of 312 ms. The ventricular rate was 81 beats/minute and the QRS duration was measured at 100 ms.

The patient’s vital signs showed a heart rate of 77 beats/minute, blood pressure of 108/67mmHg, and a temperature of 37.1°C. The physical exam was unremarkable, and the electrocardiogram (EKG) showed a sinus rhythm with a first-degree atrioventricular (AV) block (Figure 1).

**Figure 2:**
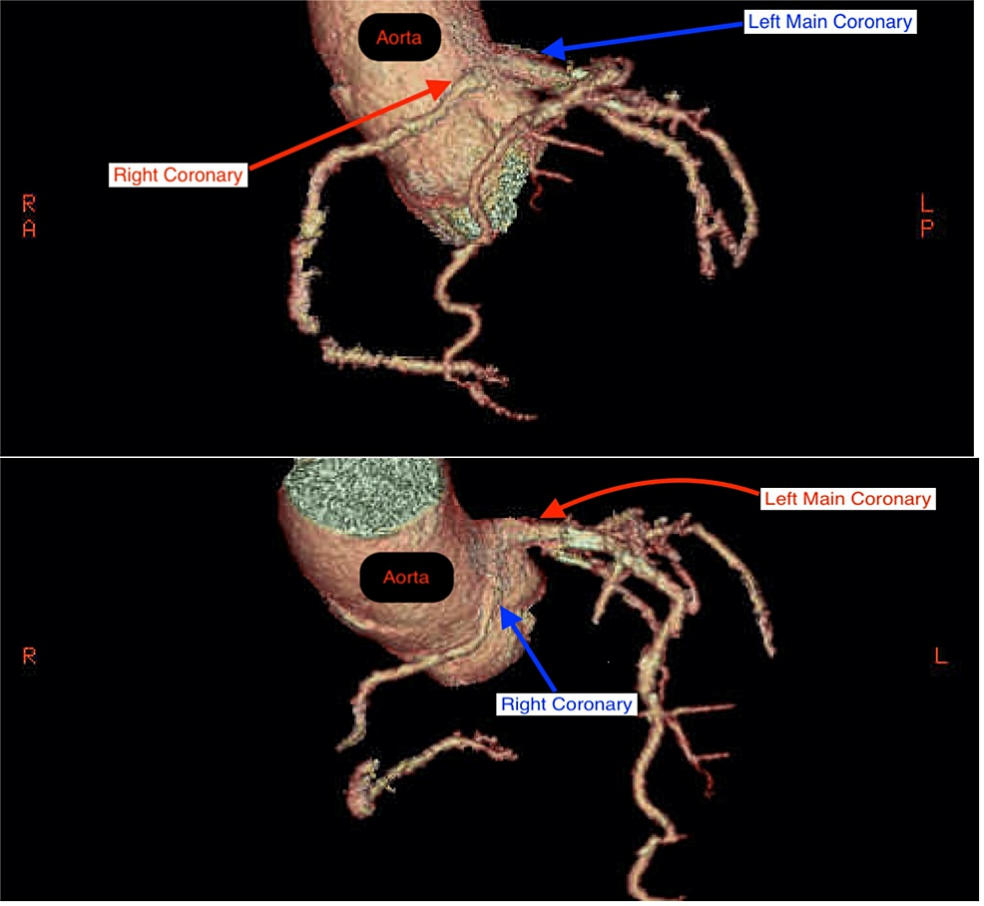
CCTA 3D reconstructed images identifying the anomalous RCA origin from the left aortic cusp and its surrounding structures. 3D: three-dimensional; CCTA: coronary computed tomography angiogram; RCA: right coronary artery

**Figure 3:**
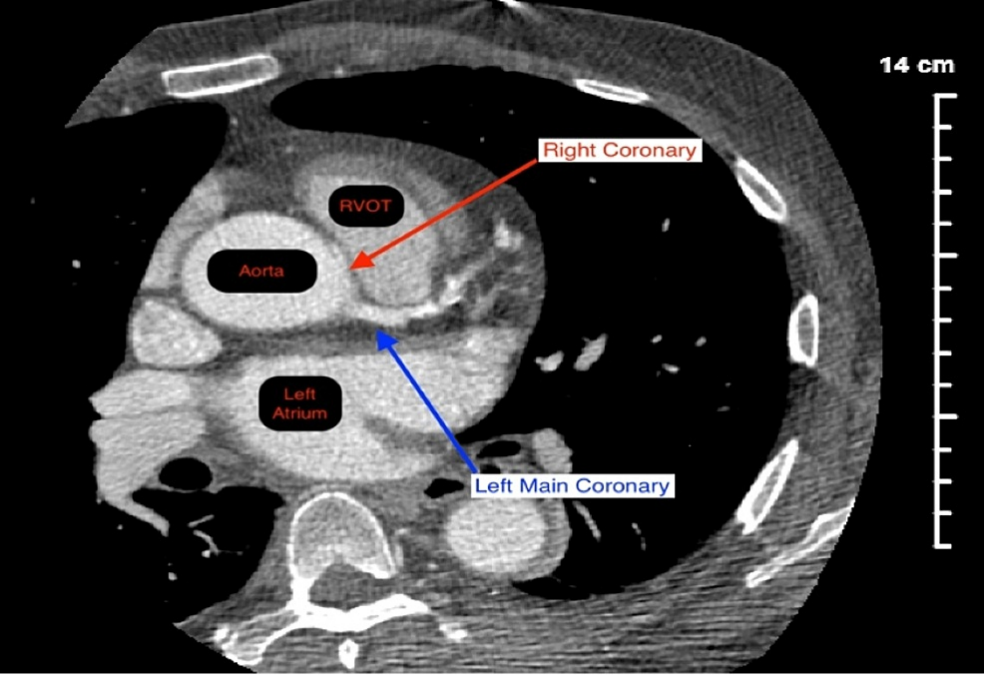
CCTA revealing an anomalous RCA with an acute takeoff angle originating from the left coronary sinus (red arrow) traveling in between the aorta and RVOT. CCTA: coronary computed tomography angiogram; RCA: right coronary artery; RVOT: right ventricular outflow tract

Laboratory testing showed a mild elevation in brain natriuretic peptide (188 pg/mL) and glucose (138 mg/dL) without an elevation in cardiac enzymes. The chest radiograph was unremarkable. A bilateral ultrasound of the carotid arteries showed minimal plaque formation without any significant stenosis or occlusion. A transthoracic echocardiogram revealed mild concentric left ventricular hypertrophy as well as trace mitral regurgitation. Next, a thallium stress test uncovered a large reversible inferior wall defect with a resting ejection fraction of 74% and a stress ejection fraction of 78%. Cardiac catheterization was done shortly thereafter which displayed significantly discrete stenosis of the obtuse marginal artery, mild stenosis of the proximal left anterior descending (LAD) artery, and an anomalous RCA. The RCA was not selectively engaged despite multiple attempts using various catheters. A follow-up coronary computed tomography angiogram (CCTA) was performed which showed a right dominant cardiovascular system with diffuse moderate stenosis of the right proximal coronary artery with an anomalous RCA originating from the left sinus of Valsalva between the aortic root and RVOT (malignant inter-arterial route) (Figures 2, 3).

The patient was treated with a coronary artery bypass graft (CABG) by means of a reversed saphenous vein graft to supply the distal RCA and a left internal mammary artery graft to the obtuse marginal artery. The postoperative course was complicated by the development of fever which prompted the initiation of a septic workup. A urinary tract infection was diagnosed and appropriately treated. The patient was monitored for the next few days and recovered well without any major complications. He was discharged on postoperative day six. The patient’s medical therapy was optimized with the addition of aspirin 81 mg to his previous medications with advice to continue the regimen at home. The patient was also recommended to follow up with a cardiac rehabilitation facility as well. At the time of his discharge, the patient was ambulating well without any constitutional or cardiac symptoms.

## Discussion

Here we report a rare case of an anomalous RCA with a malignant course between the aorta and RVOT. The prevalence of a coronary artery anomaly is difficult to define but should not be excluded from a differential diagnosis due to the potentially dangerous hemodynamic effects associated with malignant variants. The literature review in this discussion is primarily focused on malignant courses of RCA anatomy as opposed to benign variants that are clinically silent and require no intervention.

Etiology

Anomalous coronary arteries are congenital and refer to coronary vessels that have an abnormal origin site, course, and/or distribution throughout the myocardium. Given its congenital nature, it can be presumed that there is an embryological and genetic component to the formation of these vessels. The formation of the human heart in embryogenesis is complicated and requires the integration of countless genes in a time-sensitive manner. There are numerous studies linking genetic variants to the development of coronary artery disease but there is no literature currently defining a specific genetic basis for a congenital coronary artery anomaly in humans. In mice, it has been shown that the loss of function of *Tbx1* (T-box transcription factor-1) can cause anomalous coronary artery development [1]. Familial associations have also been reported suggesting a hereditary influence [2]. These anomalies have been shown to occur more often in individuals with congenital heart disease compared to those with normal cardiac anatomy [3].

Incidence and prevalence 

The incidence of any coronary artery anomaly ranges from 0.2% to 1.7% in patients who have undergone coronary angiography studies [4-7]. According to the current literature, the most frequent anomaly found on imaging is a separate origin of the LAD and the left circumflex (LCX) arteries. There is a prevalence ranging from 0.13% to 0.18% of an anomalous RCA originating from the left sinus of Valsalva (LSV) or the LAD [8,9]. Congenital coronary artery abnormalities are the second most common cause of SCD in young athletes aged 35 and under, with the first being hypertrophic cardiomyopathy [10,11].

Anomalous RCA: benign or malignant?

**Figure 4:**
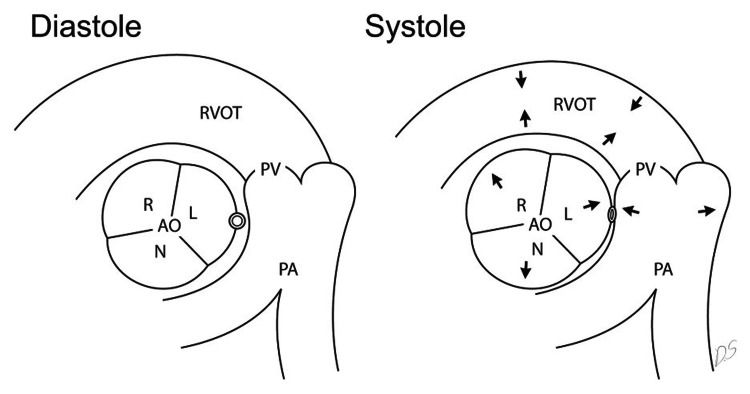
Diagram demonstrating a high interarterial course of the RCA (represented by the double circle) between the left coronary sinus and the pulmonary artery. During systole, the aorta and PA expand simultaneously compressing the RCA (as seen in the right image with the directional arrows). Adapted from Lee et al. (2010) [15]. Permission to use the figure was obtained from the Radiological Society of North America. RCA: right coronary artery; L: left coronary sinus; PA: pulmonary artery; AO: aorta; N = noncoronary sinus; PV = pulmonary valve; R = right coronary sinus

**Figure 5:**
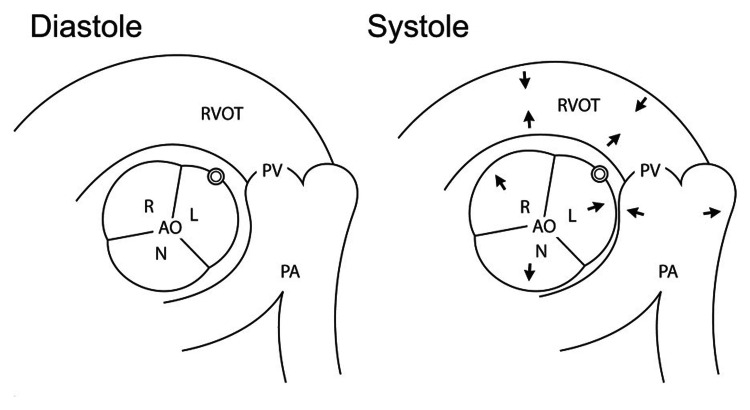
Diagram demonstrating a low interarterial course of the RCA between the aorta and RVOT. During systole, the aorta distends but the RVOT contracts resulting in less compression of the RCA when compared to a high interarterial course. Adapted from by Lee et al., (2010) [15]. Permission to use the figure was obtained from the Radiological Society of North America. RCA: right coronary artery; RVOT: right ventricular outflow tract

Multiple variations of anomalous RCAs have been previously identified. Attempts have been made to create a classification system that organizes the origin and route of anomalous coronary arteries, but there is no gold standard currently in place [12]. Benign variants are described as having retro aortic, prepulmonic, and septal (subpulmonic) courses whereas malignant variants are described as having an interarterial course [13,14]. The malignant variant can then be further divided into two subtypes: a high interarterial course (RCA between the aorta and pulmonary artery) (Figure 4) and a low interarterial course (RCA between the aorta and RVOT) (Figure 5).

The malignant courses are believed to have differing hemodynamic consequences resulting in distinct symptoms and clinical presentations which can potentially influence the management of these patients [15].

Pathophysiology of syncope and sudden death

In the setting of a malignant course of an anomalous RCA, an exact pathophysiological mechanism regarding syncope and sudden cardiac death is yet to be fully explained. Several hypotheses exist focusing on the amount of compression an aberrant RCA undergoes during ventricular systole and strenuous exercise. Coronary arteries with an acute angle takeoff and valve-like ridges at the ostium can increase the narrowing or occlusion of the vessel during aortic root expansion in systole [16]. Intravascular ultrasonography shows that in symptomatic patients with anomalous coronary arteries originating from the opposite sinus of Valsalva, lateral compression of the vessel worsened in systole [17]. An analysis of 32 necropsy cases demonstrated that coronary dominance is a substantial factor in assessing clinical significance due to increased myocardial ischemia with occlusion of the dominant vessel [18]. Age is also an important consideration when assessing the risk for SCD. According to a clinical study published in the Journal of the American College of Cardiology, researchers analyzed the records of 242 patients with isolated congenital coronary artery anomalies and found that patients aged 30 years or younger were significantly more likely to suffer from SCD during exercise in contrast to their older counterparts [19]. This is likely attributed to the fact that aortic elasticity decreases with age resulting in decreased expansion of the aorta and compression of a coronary vessel [20]. A large retrospective study involving patients with malignant anomalous RCAs originating from the left coronary sinus found that the prevalence of angina and major adverse cardiac events were significantly higher in patients who suffered from an RCA with a high interarterial course compared to a low interarterial course [15]. Anomalous coronary arteries have not yet been linked to an increased risk of the development of coronary artery disease [21]. About 27% of patients with coronary artery abnormalities have also been found to have atypical aortic root anatomy [22]. The clinical severity of coronary artery anomalies can be presumed from the collective analysis of these various factors.

Clinical presentation and diagnosis

The presentation of an anomalous RCA with a malignant course is highly variable. Clinical presenting symptoms include chest pain or discomfort, shortness of breath, palpitations, arrhythmias, syncope, and SCD. The majority of patients are asymptomatic and the diagnosis is often made incidentally or on autopsy [19,23-25]. It is important to understand that the first presentation of an anomalous RCA can be SCD with or without any associated or prodromal symptoms; therefore, it is imperative to rule out congenital abnormalities in patients presenting with these symptoms. Currently, CCTA has emerged as the gold standard imaging modality for diagnosing coronary artery abnormalities over coronary angiography as it is more effective in detecting anomalies in comparison [26,27]. The major disadvantage of using CCTA is exposure to radiation which can predispose patients to malignancy, especially in younger populations. Magnetic resonance imaging carries the benefit of no radiation exposure but is limited in its ability to assess distal vessel courses and smaller caliber vessels, as well as requiring the need for patient compliance. Echocardiograms also carry the benefit of no radiation but studies can be limited based on technician experience and possible poor acoustic window, typically in obese patients [28]. EKGs, Holter monitors, and stress testing can reveal cardiac abnormalities although they are non-specific in unveiling the etiology.

Treatment overview

Limited evidence exists that demonstrates if surgical correction of an anomalous RCA leads to a reduced incidence of SCD, especially in patients older than 35 years. There is also minimal data guiding the medical management of these patients to prevent SCD from occurring. According to American Heart Association/American College of Cardiology guidelines published in 2018, surgical intervention is a Class I recommendation in patients who experience ischemic cardiac symptoms or are shown to have ischemia during diagnostic testing [29].

Medical management

A retrospective study out of Japan reviewed 56 cases of patients with anomalous RCAs originating from the LSV who were preferentially treated medically rather than surgically. Medical management of these patients was described as limiting exercise and administering oral medications such as beta-blockers, calcium channel blockers, nitrates, and antiarrhythmic drugs. The age groups with the most promising prognosis were those in the middle to elderly aged patients. None of the studied patients had co-existing atherosclerosis [30]. Evidence supporting purely medical management is scarce and is mostly from case reports and expert opinion.

Surgical management

Several surgical interventions are available for the correction of an anomalous RCA. These include but are not limited to unroofing of the intramural segment, CABG, and reimplantation of the anomalous vessel. First introduced in 1981, surgical unroofing was utilized as a potential intervention to correct an anomalous coronary artery by Mustafa et al. [31]. This technique involves rerouting the affected vessel via a newly created ostium in the aortic wall which approximates a normal exit of the vessel from the aorta. This procedure is reserved for patients with an intermural segment of the affected vessel. Surgical unroofing has shown to be successful and is more commonly performed in patients under 20 years old [32-35]. CABG is a viable option for every patient with an anomalous RCA but is especially practical in older patients and/or those with co-existing coronary artery disease. Bypass grafting is less surgically complex when compared to the alternatives and has been performed for over half a century making it ubiquitous to cardiothoracic surgeons. CABG carries its own set of risks including graft failure and decreased patency of the grafted vessel over time; however, it does eliminate the need to open and manipulate the aorta reducing the possibility of aortic insufficiency. Coronary artery disease can complicate reimplantation or a surgical unroofing procedure in the presence of a proximal plaque making CABG an increasingly more attractive option in these patients [36,37]. Reimplantation of a coronary artery involves a surgical restoration of coronary vessel anatomy by reimplanting the affected vessel into the correct anatomical location, unlike surgical unroofing. Reimplantation avoids the risks associated with CABG and unroofing, but potential complications with this procedure include stretching or kinking of the affected artery and possible stenosis of anastomotic vessels. Vessel reimplantation can be technically demanding and time-intensive due to the extensive dissection and mobilization of vessels required to complete the surgery [36]. Table 1 presents the most commonly performed procedures for anomalous coronary arteries.

**Table 1:** Tabulated list of most performed procedures for anomalous coronary arteries. The table summarizes commonly performed surgical interventions for anomalous coronary arteries, their respective indications, risks, benefits, and/or complications. Please note, this is a summary and not a comprehensive list. References used in the creation of the table: Jaggers et al. (2008) [36], Furukawa et al. (2019) [37], and Rodefeld et al. (2001) [38].

Surgical intervention	Indication/Benefits	Risks/Complications	
Unroofing	Preferred when the anomalous coronary path is initially within the aortic wall with an extended intramural segment	Subclinical ischemic changes reported particularly in pediatric patients with an anomalous right coronary artery	
	Better short-term outcomes compared to alternative procedures		
	Relatively fewer complications compared to alternative procedures		
Coronary artery bypass grafting	Preferred in elderly patients with co-existent coronary artery disease	Decreased patency of internal mammary graft secondary to competitive flow	
	Less surgically complex to perform compared to alternatives		
		Concern of early graft failure and long-term loss of patency	
	Does not require aortic manipulation and avoids the risk of aortic insufficiency		
Reimplantation	Preferred when the anomalous coronary has a short intramural segment	Technically demanding and high risk due to extensive dissection required	
	Restores normal anatomy and addresses all high-risk features of an anomalous coronary artery		
		Possible stretching and kinking of the reimplanted artery	
		Long-term risk of circumferential anastomoses	
Pulmonary artery translocation	Patients with a single coronary ostium and no intramural component	Does not address the issue of aberrant anatomy, and unknown if additional space created is enough to relieve compressive forces on coronary	
	Preferred in patients with complicated coronary anatomy		
	Reduces the risk of myocardial ischemia as the procedure can be performed during beating-heart cardiopulmonary bypass		
		Risk of pulmonary artery stenosis	
	Reduced risk of bleeding due to reduced pulmonary artery pressure		

## Conclusions

The RCA is typically the vessel responsible for supplying blood to the sinoatrial and atrioventricular nodes of the heart as well as the inferolateral myocardium. It can be hypothesized that a brisk occlusion of this vessel can result in a sudden drop in cardiac output resulting in vasovagal syncope secondary to pacing failure. Furthermore, it can be argued that repeated bouts of RCA occlusion would lead to inferior heart wall ischemia and the development of first-degree AV block, both of which were seen in our patient. Current data has not yet elucidated an exact pathophysiological mechanism, but present hypotheses revolve around the degree of compression the vessel undergoes during ventricular systole and the takeoff angle of the vessel.

During the hospitalization of our patient, multiple cardiac assessments were performed including EKG, transthoracic echocardiogram, thallium stress test, cardiac catheterization, and CCTA. From these examinations, only the catheterization and CCTA were able to identify the anomalous RCA. The CCTA imaging modality was suitable in visualizing the anomaly concerning its origin, its course, and assisted in constructing a strategy for potential surgical intervention. Our patient was found to have moderate proximal stenosis of his RCA so the decision to perform a CABG was made by the cardiothoracic surgeon. The patient tolerated the procedure without suffering any major cardiac complications postoperatively. Repeat EKG postoperatively did not show any significant changes. The patient was discharged on postoperative day six and was referred to a cardiac rehabilitation facility. At the time of discharge, the patient denied any cardiac symptoms other than discomfort at the incision site.

There is a lack of data providing an optimal treatment strategy for patients with malignant coronary artery anomalies. While surgery seems to be the mainstay of treatment in symptomatic patients, conservative management of asymptomatic patients is inadequately described in current literature. A multidisciplinary approach involving interventionalists, cardiologists, and surgeons should be employed to assess the patient’s presentation, anatomy, and risk factors to mutually generate an individualized treatment plan.
